# Lipopolysaccharide binding protein, interleukin-10, interleukin-6 and C-reactive protein blood levels in acute ischemic stroke patients with post-stroke infection

**DOI:** 10.1186/s12974-014-0231-2

**Published:** 2015-01-23

**Authors:** Hans Worthmann, Anita B Tryc, Meike Dirks, Ramona Schuppner, Korbinian Brand, Frank Klawonn, Ralf Lichtinghagen, Karin Weissenborn

**Affiliations:** Department of Neurology, Hannover Medical School, Carl-Neuberg-Str. 1, 30623 Hannover, Germany; Department of Clinical Chemistry, Hannover Medical School, Carl-Neuberg-Str. 1, 30623 Hannover, Germany; Department of Computer Science, Ostfalia University of Applied Sciences, Am Exer 2, 38302 Wolfenbuettel, Germany; Biostatistics, Helmholtz Centre for Infection Research, Inhoffenstr. 7, 38124 Braunschweig, Germany; Center for Systems Neuroscience (ZSN), Buenteweg 2, 30559 Hannover, Germany

**Keywords:** lipopolysaccharide binding protein (LBP), interleukin-10, Interleukin-6, C-reactive protein, stroke, infection, inflammation

## Abstract

**Background:**

Ischemic stroke patients are prone to infection by stroke-induced immunodepression. We hypothesized that levels of lipopolysaccharide binding protein (LBP), interleukin-10 (IL-10), IL-6 and C-reactive protein (CRP) are early predictors for the development of stroke-associated infection.

**Methods:**

Fifty-six patients with ischemic stroke (n = 51) and transient ischemic attack (TIA) (n = 5) who presented within 6 hours after symptom onset and who were free of detectable infection on admission were included in the study. Of these, 20 developed early infections during the first week. Blood samples were taken at 6, 12, and 24 hours and at 3 and 7 days after stroke onset. Levels of LBP, Il-10, IL-6 and CRP, as well as S100B, were measured as markers of inflammation and brain damage by commercially available immunometric tests.

**Results:**

In the univariate analysis, levels of LBP, IL-10, IL-6 and CRP significantly differed between patients who developed an infection and those who did not. In the binary logistic regression analysis, which was adjusted for National Institutes of Health Stroke Scale (NIHSS) on admission, stroke subtype and S100B peak levels, as indicator of the extent of brain damage, IL-10 at 6 hours, CRP at 6 hours and NIHSS on admission were identified as independent predictors of infection (IL-10: *P* = 0.009; CRP: *P* = 0.018; NIHSS: *P* = 0.041). The area under the curve (AUC) of the receiver operating characteristic (ROC) curves in relation to the dichotomized status of the infection (infection versus no infection) was 0.74 (95% confidence interval: 0.59 to 0.88) for CRP at 6 hours, 0.76 (0.61 to 0.9) for IL-10 at 6 hours, 0.83 (0.71 to 0.94) for NIHSS on admission and 0.94 (0.88 to 1) for the combination of CRP, IL-10 and NIHSS. In a subanalysis, 16 patients with early infections were matched with 16 patients without infection according to S100B peak levels. Here, the temporal pattern of LBP, IL-10, IL-6 and CRP significantly differed between the patient groups.

**Conclusions:**

Our data show that blood levels of inflammation markers may be used as early predictors of stroke-associated infection. We propose prospective studies to investigate if the calculated cut-offs of CRP, IL-10 and NIHSS might help to identify patients who should receive early preventive antibiotic treatment.

## Background

After acute ischemic stroke, urinary tract infections or pneumonia often complicate the clinical course and worsen the outcome [1]. Infections occurring within the first week after stroke onset are regarded as stroke-associated. They are correlated with stroke severity, since, on the one hand, a severe neurological deficit may facilitate infection and on the other hand severe infections may trigger neurological worsening.

Experimental and clinical studies have shown that a pronounced anti-inflammatory response may cause a state of stroke-induced immunodeficiency [2,3] via mechanisms that include the hypothalamic pituitary adrenal axis, the sympathetic nervous system and the vagus nerve (for a review see [4,5]). Thereby, stroke patients might be at a higher risk of infections. Clinical studies found increased levels of the anti-inflammatory cytokine Interleukin-10 (IL-10) in patients with post-stroke infection [6,7]. But also pro-inflammatory mediators such as C-reactive protein (CRP) and Interleukin-6 (IL-6) are increased in ischemic stroke patients who develop an infection [6,8].

Lipopolysaccharide-binding protein (LBP) is essential for the response to bacterial lipopolysaccharides. As a type I acute phase response protein, it is also produced in acute inflammation for a review see [9]. Recently, LBP levels have been shown to be increased in stroke patients with infection [10].

Considering the worse outcome in patients with post-stroke infection, the prevention of infections by early application of antibiotics has repeatedly been discussed. So far, respective studies have shown controversial results. According to a meta-analysis that included five clinical studies, antibiotics reduced neither the frequency of dependency nor death [11]. However, early identification of patients at risk for infection by molecular markers might improve selection criteria for preventive antibiotic treatment and, thereby, treatment effects. We hypothesized that early levels of the inflammatory and anti-inflammatory markers LBP, IL-10, IL-6 and CRP are biomarker candidates for the prediction of post-stroke infections in acute ischemic stroke patients.

## Methods

### Study population

Between August 2007 and February 2009, 56 patients with acute ischemic stroke (n = 51) or transient ischemic attack (TIA) (n = 5), who were admitted to the stroke unit of the Department of Neurology at Hannover Medical School, Germany within 6 hours after symptom onset, were enrolled. The patient cohort derived from a cohort of a former study [12]. Ischemic stroke was defined as an acute-onset focal neurological deficit combined with neuroimaging evidence of cerebral infarction by cranial computed tomography (CCT) or magnetic resonance imaging (MRI). TIA was defined as a transient episode of neurological dysfunction caused by focal brain ischemia without acute infarction. Exclusion criteria were history of malignant tumour, hemorrhagic stroke, any detectable infection prior to stroke onset or any immunosuppressive treatment for example, dexametasone. Post-stroke infection was defined as any infection occurring within the first week after the event. Patients were examined for signs of infection (that is, productive cough, tachypnea, dysuria, flank pain or fever) as part of the daily ward round by the treating physician. At the beginning of clinical symptoms, a diagnostic workup was performed including clinical, laboratory and radiological examination (for example, chest radiograph or abdominal ultrasound imaging). The criteria used for diagnosis and anti-infective therapy were according to the local clinical practice. In the study population, post-stroke infection was identified (median interval from stroke onset to the diagnosis of infection was 3 days) in 20 patients.

On admission, clinical and demographic data of the patients, including age, sex, stroke etiology classified according to Trial of Org 10172 in Acute Stroke Treatment (TOAST) criteria [13], white blood cell count (WBC), creatinine, estimated glomerular filtration rate (evaluated by CKD-EPI equation), vascular risk factors (arterial hypertension, diabetes mellitus, hyperlipidemia, and smoking status) and intravenous treatment with recombinant tissue-type plasminogen activator (rt-PA) were recorded. Initial stroke severity was evaluated by the National Institutes of Health Stroke Scale (NIHSS) at admission. Clinical outcome was assessed by NIHSS at 90 days.

The study was approved by the appropriate ethics governing board (ethics committee Hannover Medical School). Patients or relatives gave written informed consent.

### Blood sampling and marker quantification

Serum, EDTA- and heparin-plasma samples were drawn from patients at 6, 12 and 24 hours and 3 and 7 days after onset of symptoms. The samples were immediately centrifuged at 1,600 × g for 15 minutes (Thermo Scientific Heraeus Multifuge 3SR plus Centrifuge). The supernatant was stored at -80°C until analysis.

All biochemical parameters were measured using commercially available CE (Communautés Européennes)-certified reagents for clinical laboratory diagnostics following manufacturers’ instructions. IL-6, CRP and S100B were determined in serum samples using the Immulite 2000 for IL-6 (two point calibration with calibrators 0116L6PL and 0116L6PH, Siemens Healthcare Diagnostics, Eschborn, Germany), the BN II nephelometric analyzer for high sensitivity CRP (hsCRP) (seven point calibration with N Rheumatology Standard SL, Siemens Healthcare Diagnostics), in the case of hsCRP values >40 mg/l the cobas6000 system for CRP (six point calibration with c.f.a.s. calibrator, Roche Diagnostics, Mannheim Germany) and the cobas e411 analyzer for S100B (two point calibration with S100 CalSet, Roche Diagnostics). LBP was measured in EDTA-plasma and IL-10 in heparin-plasma using the Immulite 2000 for LBP (two point calibration with calibrators LLBL0116 and LLBH0116, Siemens Healthcare Diagnostics) and the Immulite 1000 for IL-10 (two point calibration with calibrators LXPL0114 and LXPH0114, Siemens Healthcare Diagnostics).

The interassay precision was 6.5% for IL-6, 5.5% for Il-10, 5.0% for S100B, 4.0% for CRP, and 5.6% for LBP. The intra-assay precision was 4.5% for IL-6, 3.1% for IL-10, 2.1% for S100B, 4.6% for CRP and 3.2% for LBP.

### Statistics

Data were analyzed using SPSS software package version 11.5, SigmaPlot 11.0 and R version 3.1.1 with the package pROC [14]. Baseline characteristics are shown as percentages or median with interquartile range. The differences of demographics, clinical characteristics and blood marker levels between stroke patients with and without infection were detected by Mann-Whitney *U*-test (continuous variables) or Chi-Square-test (proportions). The binary logistic regression analysis included initial levels of IL-10, IL-6 and CRP adjusted for NIHSS on admission, stroke subtype, and peak levels of S100B, using the method of backward stepwise. Receiver Operating Characteristic (ROC) curves and the corresponding area under the curve (AUC) were computed for CRP at 6 h, IL-10 at 6 h and NIHSS on admission and also for the combination of the three markers. Random forests (RF) were used to combine the three single biomarkers. In order to avoid overfitting, scores for the ROC curves of the combination of the biomarkers were calculated on the basis of jackknife or leave-one-out method, so that the random forest did not use the corresponding patient for training. Other classifiers like linear discriminant analysis (LDA) and support vector machines (SVM) yielded similar results. In a subanalysis, for comparison of time courses of marker levels, 16 patients with infection and 16 without infection were matched according to S100B peak levels (differences in levels <20%, respectively). Between-group comparisons were analyzed by the Mann-Whitney test, and within-group comparisons between initial (6 h) and follow-up time points were analyzed by Friedman test and Wilcoxon test. For within group comparisons, after Bonferroni correction for multiple testing, a probability value ≤0.01 was considered statistically significant. The relation between marker levels was assessed by Spearman rank correlation analysis. After Bonferroni correction for multiple testing, a probability value ≤0.003 was considered statistically significant.

## Results

### Demographics

The study population consisted of 56 patients. Stroke-associated infection was diagnosed in 20 of these patients. Demographic and clinical characteristics for patients with and without infection are shown in Table 1. Here, patients differed significantly for stroke subtype, clinical severity (NIHSS on admission), clinical outcome (NIHSS at day 90), and the extent of brain damage (S100B peak levels). The infections were pneumonia (n = 9), urinary tract infection (n = 10), and cholangitis (n = 1).

**Table 1:** **Clinical characteristics of patients with and without infection**

	**Infection (n = 20)**	**No infection (n = 36)**	***P***
Female	11 (55.0)	19 (52.8)	0.873
Male^a^	9 (45.0)	17 (47.2)	
Age (years)^b^	78 (63; 83)	70 (62; 79)	0.141
Stroke subtype			0.006^c^
Cardiogenic embolism^a^	13 (65.0)	7 (19.4)	
Large artery occlusion^a^	2 (10.0)	10 (27.8)	
Lacunar infarction^a^	1 (5.0)	9 (25.0)	
Unknown^a^	4 (20.0)	10 (27.8)	
Hypertension^a^	17 (85.0)	22 (61.1)	0.062
Smoker^a^	4 (20.0)	5 (13.9)	0.551
Hyperlipoproteinemia^a^	6 (30.0)	14 (39.9)	0.506
Creatinine (μmol/L)^b^	76 (63; 87)	80 (69; 96)	0.293
eGFR (ml/min per 1.73 m^2^)^b^	78 (60; 88)	71 (60; 90)	0.918
Diabetes mellitus^a^	8 (40.0)	8 (22.2)	0.158
NIHSS on admission^b^	15 (9; 18)	3 (1; 8)	<0.001^c^
NIHSS 90d^b^	6 (3; 34)	1 (0; 3)	<0.001^c^
WBC 1d (1000/μl)^b^	8.2 (6.6; 8.8)	7.2 (6.4; 8.1)	0.074
S100B peak levels (μg/l)^b^	0.21 (0.12; 0.69)	0.11 (0.09; 0.24)	0.011^c^
i.v. rt-PA^a^	6 (30.0)	9 (25.0)	0.686

eGFR, estimated glomerular filtration rate; NIHSS, National Institutes of Health Stroke Scale; i.v. rt-PA, intravenous recombinant tissue-type plasminogen activator; WBC, white blood cell count.Data are presented as numbers (percentages) ^a^ or median (interquartile range) ^b^. *P* <0.05 was considered statistically significant ^c^.

### Levels of LBP, IL-10, IL-6 and CRP differed in patients with and without infection

In the univariate analysis, levels of LBP, IL-10, IL-6 and CRP were compared between patients with and without infection (Table 2). LBP differed significantly between 12 hours and 7 days (12 h, 24 h, 3 d: *P* <0.001; 7 d: *P* = 0.019), while IL-10 levels differed significantly during the first day (6 h: *P* = 0.002; 12 h, 24 h: *P* < 0.001). IL-6 and CRP levels were significantly different at each time point (IL-6 6 h, 12 h, 24 h, 3 d, 7 d: *P* < 0.001; CRP 6 h: *P* = 0.004; 12 h, 24 h, 3 d, 7 d: *P* < 0.001).

**Table 2:** **LBP, Il-10, Il-6 and CRP in patients with and without infection**

	**Infection**	**No infection**	***P***
	**(n = 20)**	**(n = 36)**	
LBP 6 h (μg/ml)^a^	6.37 (4.81; 8.74)	5.57 (4.79; 6.77)	0.194
LBP-12 h (μg/ml)^a^	8.29 (6.53; 10.41)	5.45 (4.77; 6.44)	<0.001^b^
LBP 24 h (μg/ml)^a^	10.95 (9.40; 15.50)	6.27 (4.99; 7.19)	<0.001^b^
LBP 3 d (μg/ml)^a^	9.83 (8.78; 16.50)	6.09 (5.14; 7.65)	<0.001^b^
LBP 7 d (μg/ml)^a^	7.93 (5.89; 13.10)	6.13 (4.83; 7.75)	0.019^b^
IL-10 6 h (pg/ml)^a^	4.0 (2.2; 6.7)	2.0 (1.5; 3.1)	0.002^b^
IL-10 12 h (pg/ml)^a^	4.2 (2.9; 6.4)	2.2 (1.6; 2.8)	<0.001^b^
IL-10 24 h (pg/ml)^a^	3.9 (3.0; 5.0)	2.3 (2.0; 2.9)	<0.001^b^
IL-10 3 d (pg/ml)^a^	2.8 (2.4; 5.5)	2.7 (2.0; 3.3)	0.109
IL-10 7 d (pg/ml)^a^	3.3 (2.5; 6.4)	2.8 (2.2; 3.5)	0.178
IL-6 6 h (ng/l)^a^	18 (8; 28)	4 (3; 8)	<0.001^b^
IL-6 12 h (ng/l)^a^	18 (10; 34)	4 (3; 9)	<0.001^b^
IL-6 24 h (ng/l)^a^	16 (8; 27)	4 (3; 8)	<0.001^b^
IL6 3 d (ng/l)^a^	14 (8; 36)	3 (3; 6)	<0.001^b^
IL-6 7 d (ng/l)^a^	14 (6; 20)	4 (3; 6)	<0.001^b^
CRP 6 h (mg/l)^a^	4.94 (1.79; 10.13)	2.50 (1.07; 3.21)	0.004^b^
CRP 12 h (mg/l)^a^	7.85 (5.16; 13.90)	2.42 (1.05; 3.84)	<0.001^b^
CRP 24 h (mg/l)^a^	19.60 (9.45; 31.83)	2.7 (1.48; 4.82)	<0.001^b^
CRP 3 d (mg/l)^a^	25.50 (11.80; 59.00)	2.24 (1.24; 5.55)	<0.001^b^
CRP 7 d [mg/l)^a^	9.97 (8.49; 50.00)	2.01 (1.19; 4.33)	<0.001^b^

Data are presented as median (interquartile range) ^a^. *P* <0.05 was considered statistically significant ^b^.

### Independent association of initial levels of IL-10 and CRP with infection

For determination if initial levels (at 6 h) of IL-10, IL-6 and CRP are independently associated with infection in ischemic stroke patients, we performed a binary logistic regression analysis that was adjusted for NIHSS on admission, stroke etiology, and peak levels of S100B. The analysis revealed that IL-10 at 6 h, CRP at 6 h and NIHSS on admission are independently associated with infection (IL-10: *P* = 0.009; CRP: *P* = 0.018; NIHSS: *P* = 0.041) (Table 3).

**Table 3:** **Independent early determinants of infection after acute ischemic stroke**

**Parameters**	**Β**	**SE**	***P*** **value**
IL-10^a^	3.328	0.461	0.009
CRP^b^	2.136	0.320	0.018
NIHSS^c^	1.192	0.086	0.041

Binary logistic regression including IL-6, IL-10 ^a^ and CRP ^b^ at 6 hours, stroke severity (National Institutes of Health Stroke Scale (NIHSS) on admission) ^c^, stroke subtype and peak levels of S100B as indicator of the extent of brain damage.

### Prediction of infection by initial CRP, IL-10 and National Institutes of Health Stroke Scale

**Figure 1:**
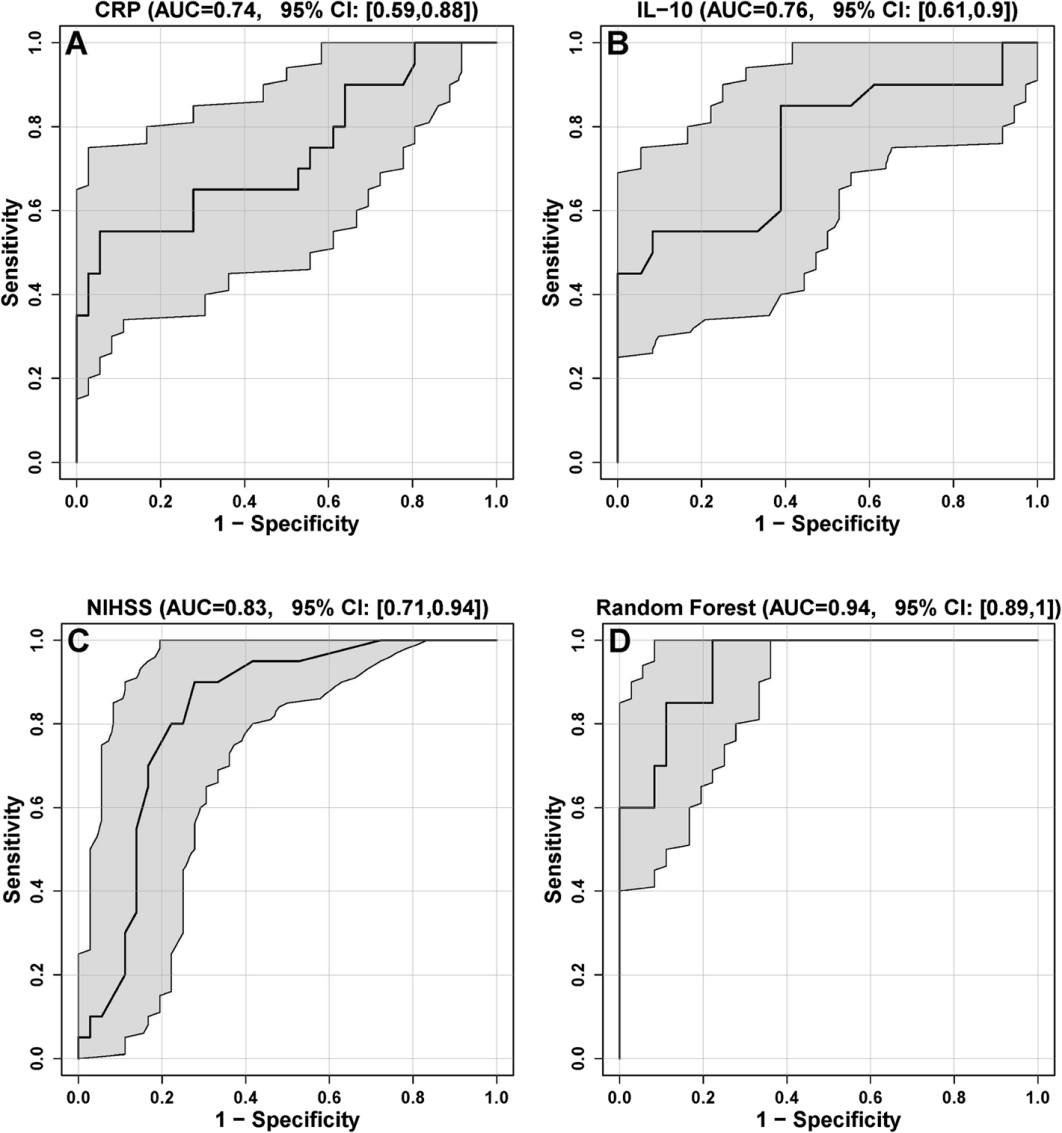
**A-D Receiver operating characteristic (ROC)-curves of initial CRP, IL-10 and National Institutes of Health Stroke Scale (NIHSS) for prediction of infection. A:** CRP: area under the curve (AUC) = 0.74 (95% confidence interval: 0.59 to 0.88), **B:** IL-10: AUC = 0.76 (95% confidence interval: 0.61 to 0.9), **C:** NIHSS: AUC = 0.83 (95% confidence interval: 0.71 to 0.94), **D:** Combination of CRP, IL-10 and NIHSS: AUC = 0.94 (95% confidence interval: 0.88 to 1) according to the method of RF (random forests) with the jackknife/leave-one-out method to compute the scores. The gray areas indicate 95% confidence regions for the ROC curves.

After identification of CRP at 6 h and IL-10 at 6 h and NIHSS on admission as independent determinants of infection, ROC analyses were performed (Figure 1. A-D). The area under the curve (AUC) for CRP in relation to dichotomized status of infection (infection versus no infection) was 0.74 (95% confidence interval: 0.59 to 0.88), 0.76 (95% confidence interval: 0.61 to 0.9) for IL-10 and 0.83 (95% confidence interval: 0.71 to 0.94) for NIHSS (Figure 1. A-C). For the combination of CRP and NIHSS the AUC was 0.86 (95% confidence interval: 0.77 to 0.96), 0.87 (95% confidence interval: 0.76 to 0.98) for IL-10 and NIHSS and 0.94 (95% confidence interval: 0.88 to 1) for CRP, IL-10 and NIHSS, when the method of RF was used (Figure 1. D). For a specificity of 97% the ROC curve-derived sensitivity was 60% for the combination of CRP, IL-10 and NIHSS. Results using the method of LDA or SVM for the combination were comparable.

### Comparison of time courses of LBP, IL-10, IL-6 and CRP in patients with and without infection

**Figure 2:**
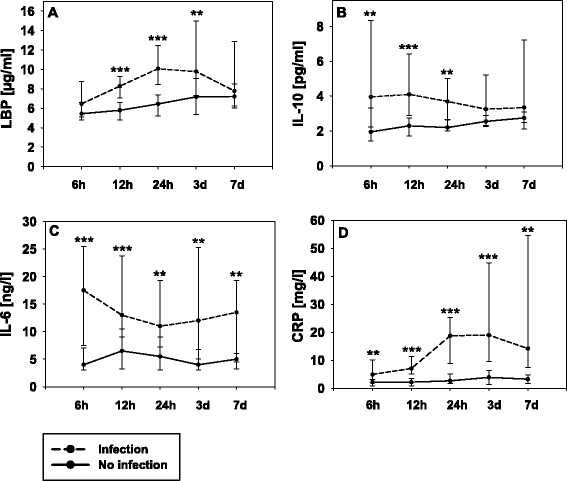
**A-D Temporal profile of LBP, IL-10, IL-6 and CRP in patients with and without infection.** Comparison of time courses between patients with (n = 16) and patients without (n = 16) infection matched for S100B peak levels for the extent of brain damage. Data are presented as median with interquartile range. Intergroup comparisons between patients with and without infection: *P ≤0.05; **P ≤0.01; ***P ≤0.001. Within-group comparisons of marker levels between initial (6 h) and follow-up time points: Patients with infection: significant differences were detected for LBP (6 h versus 12 h: *P* = 0.001, 6 h versus 24 h: *P* = 0.001, 6 h versus 3 d: *P* = 0.01) and CRP (6 h versus 12 h: *P* = 0.002, 6 h versus 24 h: *P* = 0.001, 6 h versus 3 d: *P* = 0.002). Patients without infection: significant differences were detected for LBP (6 h versus 24 h: *P* = 0.005, 6 h versus 3 d: *P* = 0.005, 6 h versus 7 d: *P* = 0.007) and CRP (6 h versus 24 h: *P* = 0.001).

For comparison of time courses of LBP, IL-10, IL-6 and CRP 16 patients with infection and 16 without infection were matched for S100B peak levels (differences in levels <20%, respectively) because the extent of the unspecific systemic inflammatory response after ischemic stroke depends on the size of brain damage. Of note, in 4 out of 20 patients with infection, no fitting matches could be identified according to S100B levels. Clinical characteristics did not differ between both patient groups (*P* >0.05). Figure 2A-D demonstrates the time courses. The temporal pattern differed significantly for each marker between the patient groups; levels in patients with infection were significantly elevated (LBP 12 h: *P* = 0.001, 24 h: *P* <0.001, 3 d: *P* = 0.007; IL-10 6 h: *P* = 0.006, 12 h: *P* <0.001, 24 h: *P* = 0.004; IL-6 6 h: *P* <0.001, 12 h: *P* <0.001, 24 h: *P* = 0.004, 3 d: *P* = 0.003, 7 d: *P* = 0.008; CRP 6 h: *P* = 0.007, 12 h: *P* <0.001, 24 h: *P* <0.001, 3 d: *P* <0.001, 7 d: *P* = 0.002).

For within-group comparisons of marker levels between initial (6 h) and follow-up time points, a significant increase was detected in patients with infection for LBP (6 h versus 12 h: *P* = 0.001, 6 h versus 24 h: *P* = 0.001, 6 h versus 3 d: *P* = 0.01) and CRP (6 h versus 12 h: *P* = 0.002, 6 h versus 24 h: *P* = 0.001, 6 h versus 3 d: *P* = 0.002). In patients without infection, marker levels significantly increased for LBP (6 h versus 24 h: *P* = 0.005, 6 h versus 3 d: *P* = 0.005, 6 h versus 7 d: *P* = 0.007) and CRP (6 h versus 24 h: *P* = 0.001).

### Correlation between LBP, IL-10, IL-6 and CRP in patients with and without infection

The correlation analysis revealed a significant correlation between LBP and CRP at 6 h (*P* <0.001), 12 h (*P* = 0.003) and 3 d (*P* <0.001), IL-10 and IL-6 at 7 d (*P* <0.001), and IL-6 and CRP at 7 d (*P* = 0.001) in patients with infection (Table 4). In patients without infection LBP versus IL-6 at 3 d (*P* = 0.003) and IL-6 versus CRP at 3 d (*P* = 0.003) were significantly correlated (Table 4).

**Table 4:** **Correlation between LBP, IL-10, IL-6 and CRP in patients with and without infection**

	**6 h**		**12 h**		**24 h**		**3 d**		**7 d**	
	**R**	**P**	**R**	**P**	**R**	**P**	**r**	**P**	**r**	**P**
**Infection (n = 16)**										
LBP versus IL-10	−0.405	0.120	−0.314	0.237	−0.284	0.286	0.100	0.712	0.041	0.879
LBP versus IL-6	−0.018	0.948	0.376	0.151	0.292	0.273	0.322	0.224	0.281	0.292
LBP versus CRP	0.879	<0.001^a^	0.697	0.003^a^	0.450	0.080	0.820	<0.001^a^	0.571	0.021
IL-10 versus IL-6	−0.014	0.959	0.221	0.411	0.309	0.244	0.399	0.126	0.785	<0.001^a^
IL-10 versus CRP	−0.393	0.132	−0.247	0.356	−0.084	0.757	0.424	0.102	0.361	0.170
IL-6 versus CRP	0.078	0.774	0.302	0.255	0.336	0.204	0.427	0.099	0.725	0.001^a^
**No infection (n = 16)**										
LBP versus IL-10	0.194	0.471	0.195	0.469	0.030	0.913	−0.233	0.384	0.183	0.498
LBP versus IL-6	0.028	0.929	0.426	0.100	0.252	0.346	0.687	0.003^a^	0.297	0.264
LBP versus CRP	0.478	0.061	0.453	0.078	0.178	0.509	0.444	0.085	0.615	0.011
IL-10 versus IL-6	−0.279	0.296	−0.037	0.893	−0.379	0.148	−0.326	0.218	0.270	0.313
IL-10 versus CRP	0.100	0.712	0.010	0.970	0.187	0.489	0.055	0.841	0.072	0.791
IL-6 versus CRP	0.280	0.293	0.402	0.122	0.407	0.117	0.689	0.003^a^	0.308	0.247

*P* ≤ 0.003 was considered statistically significant ^a^.

## Discussion

The main findings of the present study are that, in acute stroke patients, levels of LBP, IL-10, IL-6 and CRP show a different time course in patients with and without post-stroke infection and that IL-10 and CRP at 6 h and NIHSS on admission are independent predictors of stroke-associated infections.

After the acute event of stroke, concentrations of the pro-inflammatory cytokine IL-6 and the acute phase protein CRP in brain tissue and peripheral blood are increased since they are rapidly released by activated cells [12,15]. Also, in the acute stage after stroke, a strong anti-inflammatory reaction results in a suppression of the immune system [5]. As a mechanism, immunodepression is hypothesized to favor the development of infections, for example, from microaspiration to pneumonia or from asymptomatic bacteriuria to urinary tract infection. IL-10 is a major player of the cellular and molecular suppression of inflammation [16]. Our data show that levels of the pro- and anti-inflammatory markers IL-6, CRP and IL-10 differ as early as at 6 h after stroke onset between patients with and without post-stroke infection. In patients with infection, levels remained elevated until 24 h in the case of IL-10 and until day 7 in the case of IL-6 and CRP, respectively.

In contrast to this, early levels of LBP at 6 h after stroke were not significantly associated with occurrence of infection. At later time points, LBP levels were significantly elevated until day 7 in patients with infection. In addition, we showed that LBP - as an acute-phase protein – not only increases in patients with infection but also increases in patients without infection during the first day in response to the infarction. Further investigations need to show whether the increase of LBP in circulating blood directly results from the site of infarction or represents a systemic reaction from peripheral blood cells.

Over the past years, several clinical trials have investigated preventive anti-infective treatment not only for lowering the rate of infection but also for ameliorating the clinical outcome. Mechanisms that may explain why the rate of infection could directly influence neurological outcome are the potentially detrimental effects of hyperthermia, hypotension or hypoxia on neurons [17-19]. So far, only the Mannheim Infection in Stroke Study (MISS) has reached improvement for rate of infection and clinical outcome using the prophylactic mezlocillin plus sulbactam in severe stroke patients, who presented as bedridden within 24 h of stroke onset [20]. In contrast to these results, other clinical trials investigating treatment with anti-infective drugs have failed to improve outcome [21] or did not lower the rate of infection [22]. Acute ischemic stroke patients had significantly better outcome at 30 d when treated with minocycline within 24 h compared to placebo [23]. The favorable outcome after administration of minocycline has been recently confirmed in another cohort of stroke patients [24]. But it has been discussed that minocycline acts independently from its antibiotic effects via neuroprotection.

So far, it remains unclear as to whether an assessment of molecular inflammation markers or clinical variables might be useful for decision-making in preventive anti-infective treatment. Recently, the predictive ability of both, IL-6 and CRP within 3 days after stroke for post-stroke infection has been reported [25,26]. However, the time points used for the determination of inflammation markers might be too late to indicate the development of infection. In two of the clinical studies investigating preventive anti-infective treatment, the association of inflammation markers and post-stroke infections was analyzed. In the Preventive Antibacterial Therapy in Acute Ischemic Stroke (PANTHERIS) trial Klehmet *et al*. [27] showed that increased levels of IL-10 were predictive for post-stroke infections independently of preventive antibacterial treatment with moxifloxacin. Therefore, the authors concluded that IL-10 levels might indicate patients who would not respond to this treatment. Also, increased IL-10 levels within 24 h were associated with infection in patients from the Early Systemic Prophylaxis of Infection after Stroke (ESPIAS) trial, a randomized clinical trial using preventive antibacterial treatment with levofloxacin [6].

Since timing is decisive in acute stroke treatment, early administration of antibiotics in a selected cohort of stroke patients might be the key to ameliorate outcome. Therefore, in contrast to former studies, we determined very early levels of inflammation markers IL-6, IL-10, CRP and LBP (at 6 h) to analyze the association with post-stroke infection. Of note, the multivariate analysis revealed an independent association of IL-10 and CRP with infections, suggesting a strong effect of the inflammatory and anti-inflammatory reaction on the development of infections.

In a subanalysis of patients matched for S100B levels, we showed that differences in inflammation markers in patients with and without infection were independent from the extent of brain lesion. As previously shown, development of infection is associated with stroke severity [28,29]. The association of stroke severity and infection can be explained since clinical deficits result in conditions such as dysphagia or shallow respiration due to *disturbance of consciousness*. But severe stroke does not obligatorily mean large infarction, as reflected by S100B peak levels, since a small infarction in the brainstem or internal capsule is related to a severe deficit, whereas a major infarction in the temporal and occipital lobe may be associated with only a mild deficit. In the current study, we found evidence for an additional independent effect of the individual inflammatory response of any patient on the development of early infection. According to our data, we propose prospective studies to investigate if the combination of markers CRP, IL-10 and NIHSS might be useful as inclusion criteria for very early initiation of preventive anti-infective treatment.

### Limitations

However, the current study has some limitations. The number of patients studied is rather small considering the multifactorial pathology of ischemic stroke, but we collected serial blood samples in all patients in order to present the temporal pattern of the studied markers. Another limitation is that only samples from peripheral blood could be achieved. The source of increased marker levels might be either peripheral blood cells or resident cells at the site of infarction. In addition, concentrations of inflammation markers at the site of infarction may only partially be reflected in the peripheral blood. However, cerebrospinal fluid, which might better represent inflammation processes in the brain tissue, cannot be achieved repetitiously and staying on schedule for the lumbar puncture would be difficult. Also, despite exclusion of patients with history of infection prior to stroke or with early signs of infection at inclusion, it cannot be ruled out that infections with only subclinical symptoms developed prior to inclusion. Finally, larger studies need to confirm the combination of markers for independent prediction of early infection. In addition, so far it remains unclear whether biomarker measurement with kits other than the ones used in the current study could reproduce the findings.

## Conclusions

The current study shows that the temporal pattern of circulating levels of LBP, IL-10, IL-6 and CRP differs between acute ischemic stroke patients with and without post-stroke infection. IL-10 and CRP at 6 h, as well as NIHSS on admission, were identified as independent predictors of stroke-associated infection. Prospective studies are warranted to confirm these surrogate markers as independent early predictors for infection in acute ischemic stroke patients. This might help to identify patients who should receive early preventive antibiotic treatment.

## Abbreviations

AUC: area under the curve
CCT: cranial computed tomography
CRP: C-reactive protein
ESPIAS: early systemic prophylaxis of infection after stroke
IL-10: interleukin-10
IL-6: interleukin-6
LBP: lipopolysaccharide binding protein
MRI: magnetic resonance imaging
MISS: Mannheim Infection in Stroke Study
NIHSS: National Institutes of Health Stroke Scale
PANTHERIS: Preventive Antibacterial Therapy in Acute Ischemic Stroke
TIA: transient ischemic attack
WBC: white blood cell count
